# Factors influencing somatic embryogenesis, regeneration, and *Agrobacterium*-mediated transformation of cassava (*Manihot esculenta* Crantz) cultivar TME14

**DOI:** 10.3389/fpls.2015.00411

**Published:** 2015-06-10

**Authors:** Evans N. Nyaboga, Joshua M. Njiru, Leena Tripathi

**Affiliations:** Bioscience Center, International Institute of Tropical AgricultureNairobi, Kenya

**Keywords:** cassava, friable embryogenic calli, genetic transformation, co-centrifugation, transgenic plants

## Abstract

Routine production of large numbers of transgenic plants is required to fully exploit advances in cassava biotechnology and support development of improved germplasm for deployment to farmers. This article describes an improved, high-efficiency transformation protocol for recalcitrant cassava cultivar TME14 preferred in Africa. Factors that favor production of friable embryogenic calli (FEC) were found to be use of DKW medium, crushing of organized embryogenic structures (OES) through 1–2 mm sized metal wire mesh, washing of crushed OES tissues and short exposure of tyrosine to somatic embryos; and transformation efficiency was enhanced by use of low *Agrobacterium* density during co-cultivation, co-centrifugation of FEC with *Agrobacterium*, germination of paramomycin resistant somatic embryos on medium containing BAP with gradual increase in concentration and variations of the frequency of subculture of cotyledonary-stage embryos on shoot elongation medium. By applying the optimized parameters, FEC were produced for cassava cultivar TME14 and transformed using *Agrobacterium* strain LBA4404 harboring the binary vector pCAMBIA2301. About 70–80 independent transgenic lines per ml settled cell volume (SCV) of FEC were regenerated on selective medium. Histochemical GUS assays confirmed the expression of *gus*A gene in transformed calli, somatic embryos and transgenic plants. The presence and integration of the *gusA* gene were confirmed by PCR and Southern blot analysis, respectively. RT-PCR analysis of transgenic plants confirmed the expression of *gus*A gene. This protocol demonstrates significantly enhanced transformation efficiency over existing cassava transformation protocols and could become a powerful tool for functional genomics and transferring new traits into cassava.

## Introduction

Genetic transformation technology has become a high throughput platform for cultivar improvement in several crops as well as for studying gene function in plants. Despite cassava (*Manihot esculenta* Crantz) being an important staple food crop for millions of people throughout the tropics; research in the field of transgenic improvement and functional genomics in cassava is constrained by low efficiency and cultivar dependent transformation systems, and therefore an efficient transformation protocol, which does not necessitate further genotype specific standardization, is crucial for cassava genomics and improvement initiatives.

Currently, the use of friable embryogenic calli (FEC) has been considered the most preferred explants for *Agrobacterium*-mediated transformation of cassava. Several protocols using FEC as target tissues and particle bombardment or *Agrobacterium*-mediated transformation procedures have been reported (Taylor et al., 2012; Zainuddin et al., 2012; Nyaboga et al., 2013). However, establishing such embryogenic tissues is cultivar dependent and requires optimization of FEC production for each particular cultivar making it a prerequisite for agronomic improvement in that particular genetic background. Previous studies using the FEC-based cassava transformation method reported the introduction of important agronomic traits, such as reduced cyanogenesis, modified starch, and resistance to diseases (Liu et al., 2011; Vanderschuren et al., 2012). However, much progress has been made in the development of *Agrobacterium*-mediated transformation protocols for the model cultivar 60444 (Bull et al., 2009; Taylor et al., 2012). Also the available genetic transformation systems have yielded only a few dozen or no transgenic lines for farmer-preferred cultivars since the available protocols are cultivar-dependent (Zainuddin et al., 2012). Hence, the development of efficient high-throughput genetic transformation capabilities for popular cassava cultivars is required.

“TME14” is a landrace commonly grown in West, Central, and East Africa and considered as one of the most preferred cultivars in Africa. It is ranked highest by farmers, being scored excellent for plant establishment, resistance to cassava mosaic disease (CMD), early maturation, high yield, mealiness, flavor, cooking qualities, and market value (NARO Report, 2005; Alicai et al., 2010). However, its production is severely affected by cassava brown streak disease (CBSD), cassava bacterial blight (CBB), and various insect pests such as whiteflies (*Bemisia tabaci*), mealybugs (*Phenacoccus manihoti*), and green mites (*Mononychellus tanajoa* Bondar) (Alicai et al., 2007; Ntawuruhunga and Legg, 2007; Herrera-Campo et al., 2011). Developing resistant varieties through genetic engineering potentially is the most cost-effective and sustainable method of controlling diseases and pests. Such improvement initiatives demand a high-throughput cassava transformation system to produce more transgenic plants in shorter period. *Agrobacterium*-mediated transformation of TME14 has been reported, however, the efficiency was very low; only seven transgenic lines per 18 clumps of inoculated FEC were obtained (Zainuddin et al., 2012). Also this protocol was not able to be reproduced in our lab (Nyaboga et al., 2013). The low transformation efficiency for cultivar TME14, which account for more than 50% of the cultivated cassava, is unsatisfactory and much lower than that of model and some farmer-preferred cultivars (Bull et al., 2009; Taylor et al., 2012; Nyaboga et al., 2013). This suggests that standard cassava transformation protocol for generating a large number of transformants is limited to few cultivars, indicating that experimental parameters for cassava transformation have not been fully optimized for most of the farmer-preferred cultivars. As a contribution to this effort and cassava community, we set out to test combinations of parameters that have facilitated *Agrobacterium*-mediated transformation in other recalcitrant crops to improve the transformation efficiency of cassava cultivar TME14.

In the present study, we have developed an improved *Agrobacterium-*mediated transformation protocol for cassava cultivar TME14. *In vitro* culture conditions were optimized for efficient FEC induction from somatic embryos of cultivar TME14. We examined the effects of factors that favor somatic embryogenesis/production of FEC including use of DKW basal medium, wounding and washing of somatic embryos, and short exposure of tyrosine to somatic embryos. The critical point in developing an efficient transformation system is to optimize the right combination of several factors during transformation. We evaluated the effects of density of *Agrobacterium* suspension, *Agrobacterium* strains, and co-centrifugation of FEC and *Agrobacterium* cells, as these factors are known to improve transformation efficiency in other crops. To significantly improve the regeneration frequency of *Agro*-infected calli, we investigated the effect of stepwise increase in BAP concentration, explant size, addition of silver nitrate (AgNO_3_), and frequency of sub-culturing of cotyledonary-stage embryos on shoot elongation medium. We then came up with a comprehensive protocol where all these modifications were combined to attain enhanced transformation and regeneration efficiency, thus overcoming the main hurdle in genetic manipulation of cassava. This protocol yields the highest number of transgenic lines reported to date for any cassava cultivar tested including cv. 60444, which is easy to transform. Therefore, it is appropriate for large scale production of transgenic plants needed for generation of T-DNA insertion as well as for gene targeting studies.

## Materials and methods

### Plant material

Plantlets of cassava landrace TME14 were obtained from *in vitro* germplasm collection of International Institute of Tropical Agriculture (IITA), Ibadan, Nigeria. The plantlets were maintained by regular sub-culturing at 4 weeks interval as *in vitro* plantlets on basic shoot culture medium (CBM, Supplementary Table 1) at 28°C under a 16/8 h photoperiod.

### Production of somatic embryos

Somatic embryos (SE) were induced from axillary buds (AB) and immature leaf lobes (ILL) from 3 to 4 weeks old *in vitro* plantlets. Nodal explants (10 mm long) were cut and placed horizontally on petri dishes containing axillary bud enlargement medium (CAM, Supplementary Table 1) for 4 days at 28°C in the dark for production of axillary buds. The enlarged AB from the nodal explants were removed with sterile syringe needles under a binocular microscope and transferred to callus induction medium (CIM, Supplementary Table 1) using either MS (Murashige and Skoog, 1962) or DKW (Driver and Kuniyuki, 1984) as the basal medium to evaluate the effect of different basal salt mixture media on production of somatic embryos. Similarly ILL (1–6 mm) were isolated and transferred to CIM using either MS or DKW as the basal medium. The plates with AB and ILL were incubated at 28°C in the dark for 2 and 4 weeks, respectively, to induce development of primary somatic embryos. The comparative potential of somatic embryogenesis was evaluated based on both the frequency of organized embryogenic structures (OES) production for each basal medium [Frequency of OES = (total number of explants showing somatic embryogenesis/total explants cultured) ^*^ 100], and scoring of the amount of somatic embryos (SE) obtained per OES cluster on 0–5 scale, where 0 = no SE obtained, 1 = very low SE, up to 10% of the OES cluster, 2 = low SE, 11–25% of the OES, 3 = medium SE, 26–50% of the OES, 4 = high SE, 51–75% of the OES, and 5 = very high SE with mostly structures embryogenic on entire OES cluster. The somatic embryos developed were sub-cultured onto fresh medium after removing non-embryogenic callus developing around the embryos with the help of sterile syringe needles. Four weeks old SE were used for the production of FEC.

### Production and proliferation of friable embryogenic calli (FEC)

Production of FEC was performed according to the protocol described by Nyaboga et al. (2013) with several modifications. OES developed on CIM were collected onto a metallic wire mesh (pore size 1–2 mm) and crushed with the help of spatula into small pieces by passing them though the mesh. Fine pieces of OES were transferred into a 50 ml falcon tube and washed twice with double distilled sterile water followed by one wash with liquid Gresshoff and Doy (GD) medium (Gresshoff and Doy, 1974, Supplementary Table 1) containing 12 mg/l picloram and transferred to a 100 μm nylon filter mesh and blotted on sterile paper towel. After removing the excess liquid, the filter was placed on GD media supplemented with 12 mg/l picloram and 12 mg/l L-tyrosine and incubated at 28°C in dark for 7 days. The crushed pieces of OES were transferred in clusters of 8–12 from the filter onto fresh GD medium supplemented with 12 mg/l picloram and incubated at 28°C in dark. FEC were subsequently grown at 28°C under 16/8 h photoperiod and sub-cultured onto fresh media every 3 weeks.

### Regeneration of FEC

Assessment of the FEC regeneration potential was performed with 0.5 settled cell volume (SCV) of FEC tissues spread on nylon filter and cultured on embryo maturation and germination media (MSN; Supplementary Table 1) at 28°C under 16/8 h photoperiod and sub-cultured on fresh MSN media every 10 days. The cotyledonary-stage embryo were picked and transferred to cassava embryo maturation medium (CEM; Supplementary Table 1). After 10 days on CEM medium, the cotyledonary-stage embryos were transferred and placed on same medium after every 8–10 days. Germinating shoots were transferred to CBM for establishment of plantlets.

### *Agrobacterium* strains, plasmid vector, and preparation of bacterial cultures

*Agrobacterium tumefaciens* strains EHA105 (Hood et al., 1993) and LBA4404 (Hoekema et al., 1983) harboring the pCAMBIA2301 binary vector (CAMBIA, Canberra, Australia; http://www.cambia.org) was used for transformation. The T-DNA region of the plasmid pCAMBIA2301 contains the neomycin phosphotransferase gene (*npt*II) conferring kanamycin resistance to plant cells, and the reporter gene β-glucuronidase (*gus*A), in which the coding sequence is interrupted by an intron sequence (Supplementary Figure 1). Both *npt*II and *gus*A genes are driven by the CaMV35S promoter. *Agrobacterium* cultures were prepared for transformation as described earlier (Nyaboga et al., 2013).

### *Agrobacterium* inoculation and co-cultivation of FEC

#### Effect of *Agrobacterium* density for infectivity

To investigate the effect of *Agrobacterium* density, the bacterial pellet was re-suspended in liquid GD medium supplemented with 200 μM acetosyringone (Sigma Chemical Co.) adjusted to an OD_600_ of 0.1, 0.25, and 0.5 for transformation of FEC. *Agrobacterium* strain LBA4404 harboring pCAMBIA2301 was used for this treatment. Three months old FEC (0.5 ml SCV) were co-cultivated with 5 ml culture of the *Agrobacterium* in sterile 50 ml falcon tubes, mixed to disaggregate the callus tissues and incubated for 30 min with gentle shaking. After 30 min excess liquid was removed and *Agro*-inoculated FEC tissues were transferred onto a nylon filter mesh placed on sterilized paper towel for 5 min to remove excess bacteria. The filter having *Agro*-inoculated FEC was then transferred onto co-cultivation medium (GD medium supplemented with 200 μM acetosyringone) and incubated in the dark at 22°C for 3 days. After 3 days, transient *gus*A gene expression in *Agro*-infected FEC was compared based on blue stained spotted FEC.

#### Comparison of *Agrobacterium* strains for infectivity

Three months old FEC (0.5 ml SCV) were co-cultivated with two strains of *A*. *tumefaciens*, EHA105 and LBA4404 harboring pCAMBIA2301, to compare their infectivity. The ability of the strains to transfer T-DNA to the cassava FEC was compared through transient reporter gene expression.

#### Effect of Co-Centrifugation of FEC and *Agrobacterium* cells on transformation efficiency

Effect of co-centrifugation of FEC and *Agrobacterium* cells on transformation efficiency was evaluated. An aliquot of 0.5 ml SCV of FEC was suspended in GD medium containing 1.5 ml of pre-induced *Agrobacterium* suspension. Two such treatment sets were made and labeled C (centrifuged) and NC (non-centrifuged). Each set of treatments had three replicates. The NC set of FEC were immersed in *Agrobacterium* cell suspension and incubated with gentle shaking at room temperature for 30 min. The C set of FEC was co-centrifuged with *Agrobacterium* for 5 min at 1000 rpm at room temperature and then incubated with gentle shaking at room temperature for 25 min. After 3 days of co-cultivation, FEC were washed and transient *gus*A gene expression was recorded.

Following optimization, the best variable for each transformation parameters tested were combined and used for subsequent transformation experiments.

### Selection and regeneration of transgenic plants

Following the co-cultivation step, *Agro*-infected FEC were washed three times with liquid GD medium containing 500 mg/l carbenicillin and transferred to nylon filter mesh, which was placed on GD media supplemented with 250 mg/l carbenicillin and incubated for 7 days at 28°C 16/8 h photoperiod. After 7 days of recovery phase, the nylon filter was transferred to fresh GD medium supplemented with 250 mg/l carbenicillin and 30 mg/l paramomycin and kept under 16/8 h photoperiod at 28°C for 7 days. This step was repeated twice with gradually increasing the paramomycin selection to 40 and 50 mg/l. Following the FEC selection on GD media, the nylon filter with FEC was transferred to MSN medium supplemented with 250 mg/l carbenicillin and 50 mg/l paramomycin and kept under 16/8 h photoperiod at 28°C with fortnightly sub-culturing on fresh medium.

Various factors were evaluated in order to maximize the efficiency of regeneration of *Agro*-infected embryos:

#### Effect of BAP concentration on regeneration of *Agro*-infected embryos

Matured cotyledonary-stage embryos developing on selective MSN medium were transferred to shoot induction media (CEM) supplemented with 100 mg/l carbenicillin. The effect of gradually increasing concentration of BAP was evaluated on the shooting efficiency of cotyledonary-stage embryos. The cotyledons were placed on CEM medium with 0.1 mg/l BAP for 10 days followed by transfer to CEM medium with 0.4 mg/l BAP and compared with the treatment when embryos were cultured on either CEM with 0.1 or 0.4 mg/l BAP only.

#### Effect of size of explants

The effect of size and section of the cotyledonary-stage embryos was also tested for the frequency of regeneration. Each cotyledonary-stage embryo was sliced either longitudinally or transversely and cultured on CEM media with stepwise increase of BAP concentration. The entire cotyledonary-stage embryo (without cutting) was also cultured on same medium for comparison.

#### Effect of silver nitrate on regeneration of *Agro*-infected embryos

The role of silver nitrate (AgNO_3_) on enhancement and shoot formation was investigated with cotyledonary-stage embryos obtained from MSN stage. The cotyledonary-stage embryos were cultured on CEM (0.1 mg/l BAP) medium supplemented with or without AgNO_3_ (4 mg/l). After one cycle of 10 days in CEM (0.1 mg/l BAP), the embryos were transferred to CEM (0.4 mg/l BAP) medium and further sub-culturing every 10 days onto fresh CEM (0.4 mg/l BAP) medium.

#### Effect of sub-culturing period on regeneration of *Agro*-infected embryos

The effect of increasing the subculture frequency of cotyledonary-stage embryos was also evaluated to determine its effect on germination efficiency of mature embryos. The cotyledonary-stage embryos were sub-cultured at a frequency of 8 and 14 days on CEM (0.4 mg/l BAP) medium. A control experiment was used with no sub-culturing on CEM (0.4 mg/l BAP) medium. The number of germinating embryos was recorded after 4 weeks of culture to calculate the regeneration frequency. Well-developed shoots (3–5 cm long) from all the tested parameters were transferred to CBM for rooting and subsequent establishment of plantlets.

### GUS histochemical assay

Histochemical assays for GUS activity were used to evaluate the effect of parameters influencing transformation efficiency. The GUS staining of callus and various explants (leaf, stem, and root) of putative transgenic plants was performed for transient and stable expression of *gus*A gene following the steps as earlier described (Nyaboga et al., 2013).

### PCR analysis

Genomic DNA was extracted from young leaves of *in vitro* putative transgenic lines generated using DNAeasy plant mini kit (Qiagen, GmbH, Germany). The presence of *gus*A and *npt*II genes in the plant genome of the randomly selected putatively transgenic lines was confirmed by PCR analysis using gene-specific primers. The primer sequences for the *gus*A gene were: forward 5′-TTTAACTATGCCGGGATCCATCGC-3′ and reverse 5′-CCAGTCGAGCATCTCTTCAGCGTA-3′; and the *npt*II gene: forward 5′-GGGTGGAGAGGCTATTCGGCTATGA-3′ and reverse 5′-ATTCGGCAAGCAGGCATCGC-3′, corresponding to a 528 and 542 bp amplicon, respectively. Plasmid DNA of pCAMBIA2301 and non-transformed plant DNA were used as positive and negative controls, respectively. The reaction conditions for both primer pairs were 95°C for 5 min; 35 cycles of 95°C for 30 s, 62°C for 40 s, and 72°C for 50 s; and a final 10-min extension at 72°C. PCR products were analyzed by gel electrophoresis on 1% (w/v) agarose gels stained with GelRed™ (Biotium) and visualized under a UV transilluminator.

### Southern blot analysis

Southern blot analysis was carried using the DIG DNA Labeling and Detection Kit (Roche Applied Sciences, Mannheim, Germany) according to the manufacturer's instructions. Genomic DNA was isolated from randomly selected PCR-positive *in vitro* grown plants using cetyltrimethylammonium bromide (CTAB) method (Soni and Murray, 1994). About 20 μg of genomic DNA was digested overnight at 37°C with *Hind*III (New England Biolabs, USA) for which there is a single recognition site in the T-DNA of pCAMBIA2301, separated by electrophoresis on 0.8% (w/v) agarose gel at 40 V for 6 h, and transferred to Hybond-N+ nylon membrane (Roche) and fixed by cross-linking in a STRATA-LINK™ UV cross-linker. The blots were hybridized with digoxigenin (DIG)-labeled *gus*A-specific probe generated using a PCR DIG Probe Synthesis Kit (Roche Applied Sciences, Mannheim, Germany). Hybridization and detection were carried out using a DIG Luminescent Detection Kit for Nucleic Acids (Roche Applied Sciences, Mannheim, Germany), according to the manufacturer's instructions.

### RNA extraction and reverse transcriptase (RT)-PCR

To examine transgenes expression, total RNA was isolated from 100 mg young leaf tissue of nine randomly selected transgenic lines and non-transgenic control plant using the RNeasy plant mini kit and on-column DNase treatment according to the manufacturer's protocol (Qiagen, GmbH, Hilden, Germany). RNA quantity and purity were determined using a NanoDrop ND-2000 spectrophotometer (NanoDrop products, Wilmington, USA). Only RNA samples with A_260/280_ ratios between 2.1 and 2.2 were further processed. RNA was checked with PCR for absence of genomic DNA. The first strand cDNA synthesis was performed using 2 μg of total RNA and reverse transcriptase of the Maxima H Minus First Strand cDNA synthesis kit with oligo (dT)_18_ primers (Thermo scientific, Waltham, MA) according to the manufacturer's instructions. RT-PCR was performed with 2 μl of each cDNA synthesized using primers specific to the *gus*A gene (primer sequences as described above). Protein phosphatase 2A (*PP2A*) amplification using gene-specific primers: forward 5′-TGCAAGGCTCACACTTTCATC-3′ and reverse 5′-CTGAGCGTAAAGCAGGGAAG-3′ was used as internal control to check the quality of synthesized cDNA. The amplified RT-PCR products were separated by gel electrophoresis on 1% (w/v) agarose gels stained with GelRed™ (Biotium) and visualized under a UV transilluminator.

### Statistical analysis

All the experiments were conducted in triplicate. Each parameter for regeneration and transformation was tested in triplicates and repeated three times. The data were assessed by analysis of variance using GenStat. Data for the optimization experiments were analyzed using One-Way ANOVA and differences among treatment means were analyzed by Duncan's multiple-range test (DMRT) at a 95% confidence level (*p* < 0.05).

## Results and discussion

Cassava remains popular staple food crop among resource poor farmers mostly due to its resilience and capacity to grow on marginal lands, and its flexible harvest period, which can be as long as 30 months after planting. It is, however, relatively low in nutrients and susceptible to several pests and diseases, including attacks from whitefly, mealybug, the widely-spread green mite, CBB, CMD, and CBSD, which significantly reduce cassava yields and pose a constraint to poor farmers with little or no response capacity (Herrera-Campo et al., 2011). The lack of resistance genes in the available germplasm, high heterozygosity, allopolyploidy, low fertility, and unsynchronized flowering make cassava improvement by conventional breeding a long and tedious process (Ceballos et al., 2004; Rudi et al., 2010). Therefore, cassava genetic engineering has emerged as a valuable alternative and complementary approach to improve cassava (Liu et al., 2011; Sayre et al., 2011). However, to be successful, these applications require efficient plant regeneration and transformation protocols for most agronomically important cassava cultivars. Development of such protocols particularly for farmer preferred cultivars required an in-depth study of factors affecting somatic embryogenesis, FEC production, *Agrobacterium*-mediated transformation and regeneration efficiency.

### Production of somatic embryos (SE) and friable embryogenic calli (FEC)

**Figure 1:**
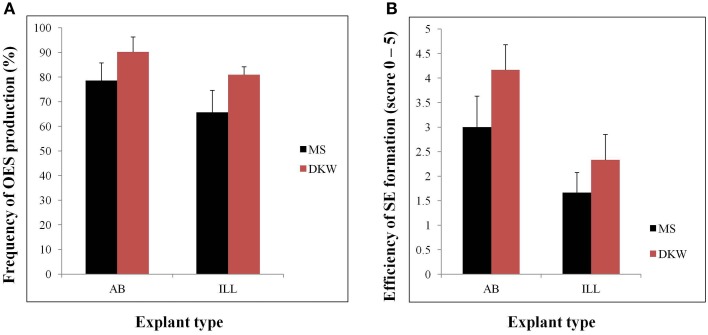
**Organized embryogenic structures (OES) and primary somatic embryos (SE) produced by different types of explants (AB and ILL) on MS or DKW medium supplemented with 12 mg/l picloram. (A)** Frequency of OES production from different type of explants cultured on MS or DKW basic medium. OES production frequencies were recorded by calculating the ratio of the total number of explants showing somatic embryogenesis/total cultured explants ^*^100. **(B)** Efficiency of SE production from AB and ILL explants. Scores are on a scale of 0–5 where 0, no SE obtained; 1, very low SE, up to 10% of the OES cluster; 2, low SE, 11–25% of the OES; 3, medium SE, 26–50% of the OES; 4, high SE, 51–75% of the OES; and 5, very high SE with mostly structures embryogenic on entire OES cluster.

Efficient tissue culture systems are essential for the production of transgenic plants. With somatic embryogenesis forming the basis of all existing genetic transformation systems for this crop, research in our laboratory has focused on development of improved and highly efficient systems for the induction and manipulation of this process in a popular cassava cultivar TME14. OES and SE were obtained from both immature leaf lobe (ILL) and axillary bud (AB) explants cultured on either DKW or MS based medium supplemented with auxin picloram. The explant source (ILL or AB) as well as the basal media (DKW or MS) had significant (*p* ≤ 0.05) effects on the frequency of OES and efficiency of SE production (Figure 1). High frequencies of OES (90.19% and 80.15% for AB and ILL, respectively) were formed when explants were cultured on DKW as compared to MS media (Figure 1). Also the frequencies of OES were high when AB was used as explants with both in DKW and MS based media compared with ILL (Figure 1). The formation of SE was observed after 12–16 and 24–28 days using AB and ILL explants, respectively. The highest scores (3.0–4.2) for SE production was obtained from AB on both media in comparison to ILL (1.7–2.3), demonstrating significantly higher efficiency of SE production from AB compared with ILL (Figure 1). The AB explants was used in further experiments as it had not only generated more primary SE, but also produced low quantities of non-embryogenic callus in comparison to ILL explants.

Use of DKW based media significantly increased the frequency of OES and SE production compared to MS medium from both AB and ILL (Figure 1). In all previous cassava transformation protocols reported, somatic embryos have been induced by culture of ILL or AB on MS based medium supplemented with auxin (Taylor et al., 2012; Vanderschuren et al., 2012; Zainuddin et al., 2012; Nyaboga et al., 2013). This study reports improved production of SE by using DKW basal media. Since, SE formation was found to be most efficient using DKW medium and AB as the explant in comparison to ILL, initiation of all the subsequent tissue culture for this investigation used DKW medium and AB explants.

Regular production of high quality FEC for use in *Agrobacterium*-mediated transformation experiments is essential for sustained production of transgenic plants. However, FEC induction strongly depends on the genotype (Liu et al., 2011). Several laboratories have made unremitting efforts by investigating various genotypes for FEC production, and success has been achieved in limited number of cultivars, such as TME1, TME7, TME14, T200, Serere, Ebwanatereka, TMS91/02327, Kibandameno, Rosinha, and Adira (Ibrahim et al., 2008; Zainuddin et al., 2012; Chetty et al., 2013; Nyaboga et al., 2013), although the efficiency of transformation is very low (only few dozen transgenic lines regenerated) and the protocol is not reproducible in different labs for example TME14 (Nyaboga et al., 2013). In all these previous studies FEC is induced from the SE over three or more subculture cycles on GD supplemented with 12 mg/l picloram (Bull et al., 2009; Taylor et al., 2012; Zainuddin et al., 2012). In our previous study, transfer of somatic embryogenic structures of TME14 to GD medium following the previously reported protocols did not result in production of FEC (Nyaboga et al., 2013). FEC production was attempted in about 20 different induction experiments but none was successful. The consequence was that all attempts over a period of one and half years failed comprehensively to deliver any FEC tissues, despite daily and on-going experiments. The requisite for a reliable FEC production protocol for this cultivar generally evolved into an extensive re-evaluation of the procedure.

**Figure 2:**
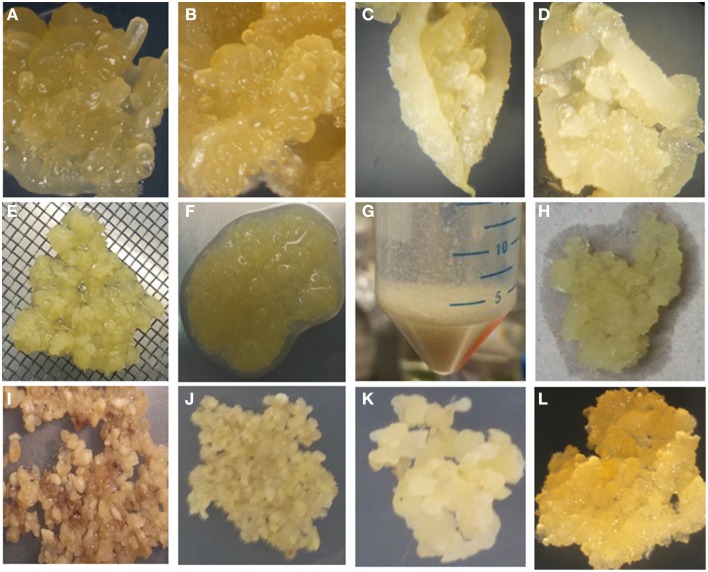
**Improved tissue culture steps for production of FEC of cultivar TME14. (A)** Somatic embryos produced on MS medium using AB, **(B)** somatic embryos produced on DKW medium using AB, **(C)** somatic embryos produced on MS medium using ILL, **(D)** somatic embryos produced on DKW medium using ILL, **(E)** crushing of somatic embryos through 1–2 mm size metal wire meshes, **(F)** crushed somatic embryos tissues, **(G)** washing of crushed somatic embryos in falcon tubes, **(H)** blotting of crushed somatic embryos after washing to remove liquid, **(I)** crushed somatic embryos tissues without washing and blotting spread evenly on GD medium supplemented with 12 mg/l tyrosine for 7 days, **(J)** crushed pieces of somatic embryos, washed, blotted and spread evenly on GD medium supplemented with 12 mg/l tyrosine for 7 days, **(K)** clusters of somatic embryos pieces on GD medium supplemented with 12 mg/l picloram, **(L)** proliferating FEC on GD medium.

It has been reported previously that wounding of SE encourages FEC production (Taylor et al., 2012). In this study, rapid processing of the SE to produce FEC was improved by crushing the SE through 1–2 mm size metal wire mesh and washing with sterile water and GD media before culturing on GD medium containing 12 mg/l picloram and 12 mg/l L-tyrosine for 7 days and then transferring onto same media but without L-tyrosine for 3 weeks. Small groups of FEC became visible developing from the clusters of meshed SE within 3 weeks of culture on GD medium. This initial FEC was selectively removed from the non-embryogenic callus and used to establish the second cycle on GD containing 12 mg/l picloram and the process repeated 3 weeks later to generate a third cycle on the same medium, generating homogenous FEC suitable for transformation (Figure 2). These improvements resulted in a large amount of FEC production in three cycles of 21 days each on GD in comparison to six cycles for other farmer-preferred cultivars in previous reports (Nyaboga et al., 2013). However, crushed SE cultured on GD medium containing 12 mg/l picloram for 7 days (with or without tyrosine exposure) without washing turned brown and necrotic and did not result in FEC production after subsequent transfer to the same media for 3 weeks (Figure 2).

This protocol has been adopted in our lab for production of FEC of cultivar TME14 and even persons who had limited experience of tissue culture were able to generate FEC of moderate quality that could still be transformed and regenerated into plants. The factors that led to FEC production in TME14 compared to Nyaboga et al. (2013) includes the use of DKW medium, crushing of OES through 1–2 mm sized metal meshes, washing of the crushed OES structures and short exposure of tyrosine to somatic embryos.

### Effect of cell density of *Agrobacterium* culture on transformation efficiency

The ratio between *Agrobacterium* and host cell density is an important factor that influences T-DNA transfer frequency; low concentration of *Agrobacterium* may reduce the efficacy of T-DNA transfer, while high concentration of bacteria may affect the viability of plant cells (Wang et al., 2009). The effect of *A. tumefaciens* concentration on transient expression of *gus*A gene in *Agro*-infected FEC tissues was determined using three different cell densities. The effect of bacterial densities on transformation was significantly (*p* = 0.05) different among OD_600_ of 0.1 and 0.25, whereas it was not different for 0.25 and 0.5 (Table 1). Of the cell optical densities tested, the OD_600_ of 0.1 yielded the lowest frequency of GUS-positive calli (25.59%) in comparison to the OD_600_ of 0.25 (63.63%) and 0.5 (66.31%) (Table 1).

**Table 1:** **Effect of *Agrobacterium* concentration, strains and co-centrifugation on transient GUS expression and number of cotyledonary stage embryos produced on selective medium**.

**Factors**	**Treatments**	**Frequency of transient GUS expression (%)**	**Average no. of cotyledonary stage embryos on selective medium**
*Agrobacterium* concentration	LBA4404 at OD_600_ of 0.1	25.59±4.17^d^	59.67±4.16^d^
	LBA4404 at OD_600_ of 0.25	63.63±4.73^b^	131.33±8.02^b^
	LBA4404 at OD_600_ of 0.5	66.31±2.50^b^	71.33±9.29^c^
*Agrobacterium* strains	LBA4404 (OD_600_ of 0.25)	65.38±4.12^b^	137.45±7.32^b^
	EHA105 (OD_600_ of 0.25)	64.29±5.33^b^	133.22±11.21^b^
Co-centrifugation	Centrifugation (LBA4404, OD_600_ of 0.25)	91.22±4.48^a^	153.67±8.62^a^
	No centrifugation (LBA4404, OD_600_ of 0.25)	62.28±3.96^b^	130.33±11.02^b^

*The values represent means ± SD, each from three independent experiments. Within each factor, means followed by different letters are significantly different (p ≤ 0.05) according to Duncan's multiple range test (DMRT)*.

To determine the lowest but most effective *Agrobacterium* concentration suitable for regeneration of transgenic plants, we further investigated the effect of different *Agrobacterium* cell densities on the number of matured cotyledonary-stage embryos produced. *Agrobacterium* concentration of OD_600_ of 0.25 produced a significantly (*p* = 0.05) higher number of cotyledonary-stage embryos as compared with OD_600_ of 0.5 (Table 1). Based on *gus*A expression and production of cotyledonary-stage embryos, OD_600_ of 0.25 was selected for genetic transformation of cassava cultivar TME14. In cassava transformation, the effect of *A. tumefaciens* cell density (OD_600_) on transient expression of *gus*A and transformation of cassava has not been reported in previous studies (Bull et al., 2009; Taylor et al., 2012; Zainuddin et al., 2012; Nyaboga et al., 2013). In other plant species, the effect of *Agrobacterium* density on transformation efficiency is dependent on the species and/or cultivar (Dutt and Grosser, 2009). Therefore, the selection of optimum *Agrobacterium* cell density and suitable plant cultivar might be necessary to increase the transformation efficiency. The results of the present study showed that the optimal *Agrobacterium* concentration suitable for transformation of cassava cultivar TME14 is OD_600_ of 0.25. These results are however contradictory to previous reports where an OD_600_ of 0.5 was recommended as an optimal *Agrobacterium* concentration for transformation of cassava (Bull et al., 2009; Taylor et al., 2012; Zainuddin et al., 2012; Nyaboga et al., 2013).

### Effect of *Agrobacterium Tumefaciens* strains on transformation efficiency

*Agrobacterium* strains play an important role in the transformation process, as they are responsible not only for infectivity but also for the efficiency of gene transfer (Bhatnagar and Khurana, 2003). The virulence of *Agrobacterium* strains varies widely among host plant species depending on the interaction between the *Agrobacterium* strain and host plant (Davis et al., 1991). So far *Agrobacterium* strain LBA4404 has been the only one used for cassava transformation (Bull et al., 2009; Taylor et al., 2012; Nyaboga et al., 2013) and the effect of *Agrobacterium* strains on cassava transformation has not been reported. In this study, we tested the efficiency of gene delivery using *Agrobacterium* strains, LBA4404 (Hoekema et al., 1983) and EHA105 (Hood et al., 1993). There was no significant difference between LBA4404 and EHA105 in both transient expression of *gus*A and number of antibiotic-resistant cotyledonary-stage embryos produced (Table 1). These results show that both *Agrobacterium* strains are suitable and efficient for transformation of cassava cultivar TME14. We used LBA4404 for transformation in the subsequent experiments.

### Effect of Co-Centrifugation of FEC and *Agrobacterium* cells on transformation efficiency

Co-centrifugation has been used to enhance *Agrobacterium*-mediated transformation of banana embryogenic cell suspensions (Khanna et al., 2004; Tripathi et al., 2012). In the present investigation, a dramatic effect on the transformation efficiency of cassava FEC with *Agrobacterium* was observed following the use of centrifugal force for propelling the *Agrobacterium* cells into the cassava embryogenic calli. FEC co-centrifuged with *Agrobacterium* cells showed approximately 1.5-fold increase in transient expression of *gus*A gene as compared to the non-centrifuged treatment (Table 1). The observed increase in transient gene expression and stable transformation following co-centrifugation probably results from improved contact with host cells hence providing efficient delivery of T-DNA into cells as previously reported (Khanna et al., 2004). No previous study has been reported on the effect of co-centrifugation on the transformation efficiency in cassava.

### Optimization of regeneration of transgenic plants

The efficient germination of cotyledonary-stage embryos into plantlets is also an important step for whole plant regeneration and mass proliferation. The ability to regenerate complete plants from transformed cotyledonary-stage embryos is the main bottleneck of achieving high transformation efficiencies as observed in our previous study (Nyaboga et al., 2013). Low transformation efficiency in cassava due to poor germination of cotyledonary-stage embryos has also been reported in studies from other laboratories. Ihemere et al. (2006) reported development of only 26 transgenic plantlets from more than 872 paramomycin resistant cotyledonary-stage embryos from cassava genotype TMS71173. Similarly, Chetty et al. (2013) obtained only 33 transgenic lines from 514 hygromycin-resistant cotyledonary-stage embryos for cassava cultivar T200. In this study, preliminary experiments were performed to investigate the possibility of improving the germination efficiency of cultivar TME14 by evaluating the effect of gradual increasing concentrations of BAP, explant size, addition of silver nitrate (AgNO_3_) and frequency of cotyledonary-stage embryos sub-culture to fresh media. The shoot formation was increased to 70.0% when cotyledonary-stage embryos were cultured on medium supplemented with 0.1 mg/l BAP for 2 weeks, followed by a transfer to the fresh medium supplemented with 0.4 mg/l BAP for 2–4 weeks, in comparison to when cotyledonary-stage embryos were cultured continuously on 0.1 or 0.4 mg/l BAP for 6 weeks for shoot induction and elongation step. These results indicate that low level of BAP (0.1 mg/l) was probably needed for the initiation of organ differentiation and thereafter a high concentration of BAP (0.4 mg/l) for shoot formation from cotyledons.

It has been reported from studies in different crops (such as sunflower, soybean, melon) that regeneration efficiency varies for the different sections of the cotyledonary region used (Leshem, 1989; Knittel et al., 1991; Baker et al., 1999; Vega et al., 2006). These studies found that when cotyledons were excised and divided perpendicularly to the long axis into proximal and distal sections, the proximal explants presented greater regeneration potential than distal ones. These differences in regeneration ability among regions from one cotyledon were attributed to the differential number of competent cells along the explants and/or the unequal distribution of endogenous growth regulators. In this study, the size and section of the cotyledonary-stage embryos was tested to check if it had an effect on the frequency of regeneration. Each cotyledonary-stage embryo was sliced either longitudinally or transversely and cultured on CEM media with stepwise increase of BAP concentration. The frequency of regenerating cotyledonary-stage embryos and the number of shoots per cotyledon were affected by the size and section of cotyledonary-stage embryos used (Table 2). Longitudinal or transverse slicing of the cotyledon-stage embryos had negative effect on both the shooting efficiency and number of shoots regenerated per embryo (Table 2). The cotyledon-stage embryos devoid of lamina showed low regeneration frequency, as well as significant reduction in the number of shoots per cotyledon-stage embryo. These results showed that the cells capable of developing into shoots are located at the base of cotyledonary-stage embryo, but for expression of their full potential they require some unknown diffusible morphogenetic factor from lamina. This result could also be attributed to the fact that longitudinal/transverse slicing of cotyledonary-stage embryos increased wound surface, which resulted in production of ethylene that negatively affected their regeneration ability (Kumar et al., 2009). Hence, whole cotyledonary-stage embryos were more efficient in germination and were used for regeneration of shoots in further experiments.

**Table 2:** **Effect of BAP concentration, size of cotyledonary-stage embryos, silver nitrate and frequency of cotyledonary-stage embryos sub-culturing on efficiency of *in vitro* germination and regeneration of plantlets of cassava cultivar TME14**.

**Parameter tested**	**Treatment**	**Germination effeciency (%)**	**Mean no. of shoots per cotyledonary stage embryos**
BAP concentration	BAP, 0.1 mg/l for 2 weeks followed by 0.4 mg/l for 2–4 weeks	70±6.67^b^	2±1.00^b^
	BAP, 0.4 mg/l for 2–6 weeks	40±3.33^d^	1.33±0.58^c^
	BAP, 0.1 mg/l for 2–6 weeks	41±4.27^d^	1.03±0.35^c^
Size of cotyledonary staged embryos	Entire cotyledonary staged embryos	68.89±6.94^b^	2±1.00^b^
	Longitudinally sliced cotyledonary staged embryos	36.67±6.67^e^	1±0.00^c^
	Transverse proximal half of cotyledonary staged embryos	46.67±3.33^c^	1.33±0.57^c^
Silver nitrate	CEM (No AgNO_3_ added)	71.11±6.94^b^	1.67±1.15^b^
	CEM supplemented with 4 mg/l AgNO_3_	67.78±8.39^b^	2.67±0.58^a^
Sub-culturing frequency	8 days	81.11±1.92^a^	2±1.00^b^
	14 days	68.89±3.85^b^	1.67±0.57^b^
	No sub-culturing till shoot formation	43.29±5.33^d^	1.13±0.33^c^

*Each treatment contained 30 cotyledonary-stage embryos and experiments repeated three times and the data are means ± SD. Within each factor, means followed by different letters indicate significant differences at p ≤ 0.05 according to DMRT*.

Silver nitrate has been shown to be effective in improving somatic embryogenesis and plant regeneration in a number of crop species including *Brassica* spp. (Eapen and George, 1997; Kuvshinov et al., 1999), maize (Carvalho et al., 1997), cowpea (Brar et al., 1999), peanut (Pestana et al., 1999), and barley (Castillo et al., 1998). As Ag^+^ ions can prevent a wide variety of ethylene-induced plant responses, including growth inhibition and senescence, the effect is assumed to be mediated through the inhibition of the physiological action of ethylene (Beyer et al., 1984), a potential inhibitor of many plant regeneration systems (Kong and Yeung, 1994). In this study, we tested the effect of silver nitrate (AgNO_3_) on regeneration of transformed embryos (Table 2). The supplementation of AgNO_3_ on regeneration media caused a significant increase in number of shoots per cotyledon but the germination efficiency of the cotyledons was not affected (Table 2) indicating that low regeneration efficiency of transformed embryos might not be due to ethylene production by cassava tissue in sealed tissue culture vessels.

Effect of sub-culture frequency on germination of cotyledonary-stage embryos was also investigated. It was found that frequent sub-culturing cotyledonary-stage embryos to fresh CEM media after every 8 days played a significant role in shooting of cotyledons (Table 2) when compared to sub-culturing every 14 days and no sub-culturing throughout the shooting period. The increase in frequency was beneficial to regeneration as there was an increase in the shoot development from cotyledonary-stage embryos. It is assumed that keeping cotyledonary-stage embryos on same medium for long leads to lose of germination potential.

### The validation and performance of the optimized transformation system

**Figure 3:**
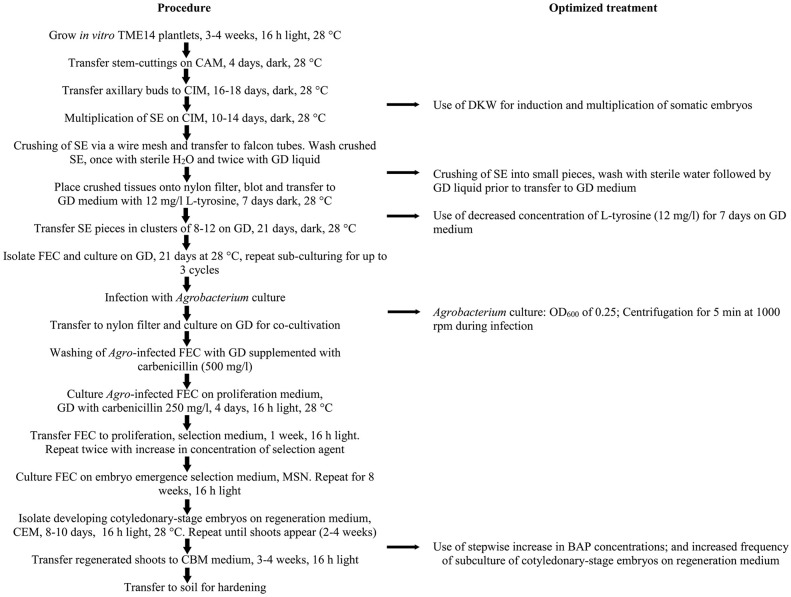
**Schematic workflow of the protocol for production of FEC and *Agrobacterium*-mediated transformation of cassava cultivar TME14**. The flow chart shows the essential steps optimized and followed in the protocol.

Based on the above results, an optimized transformation system for cassava cultivar TME14 was developed (Figure 3) and further validated by large scale experiments. The system consisted of production of FEC for cultivar TME14, centrifugation, *Agrobacterium* strain LBA4404 with OD_600_ of 0.25, a 3-days co-cultivation period, selection on 50 mg/l paramomycin and regeneration on media with a stepwise increase of BAP concentration and sub-culture of cotyledonary-stage embryos after every 8 days. The protocol developed was reproducible as no significant (*p* ≤ 0.05) difference was observed in three different transformation experiments conducted and average of about 180–199 transgenic lines were generated from 2.5 ml SCV of *Agro*-infected FEC (Table 3).

**Table 3:** **Generation of transgenic lines of cassava cultivar TME14 using *Agrobacterium* strain LBA4404 harboring pCAMBIA2301 following the optimized protocol**.

**Experiment**	**Total no. of cotyledonary-stage embryos produced on selective medium^a^**	**Germination efficiency (%)^b^**	**Regenerated transgenic lines^c^**
A	240	75.78	180
B	244	82.93	199
C	229	79.73	181

*Approximately 2.5 ml SCV of different FEC lines was used in each experiment*.

a *Total number of putatively transformed cotyledonary-stage embryos emerging from 2.5 ml SCV of FEC on selective medium supplemented with 50 mg/l paramomycin*.

b *Calculated as the number of shoots obtained on CEM divided by the total number of transformed cotyledonary-stage embryos cultured*.

c *Total number of transgenic lines generated from 2.5 ml SCV of FEC*.

**Figure 4:**
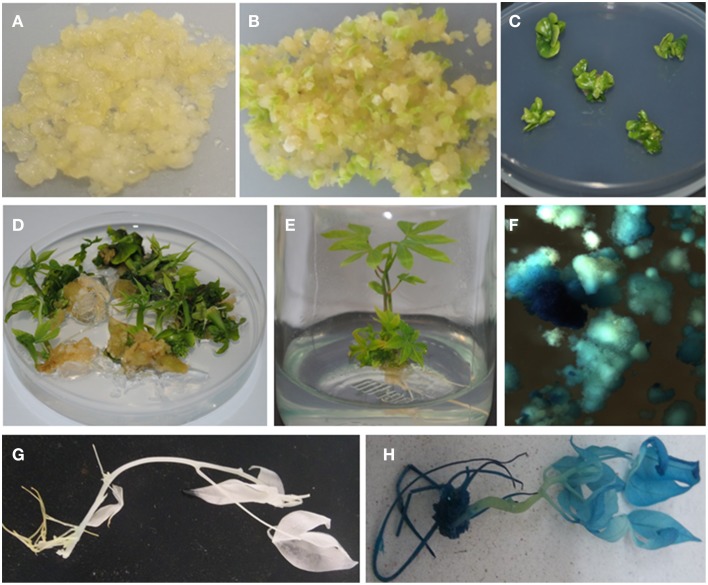
***Agrobacterium*-mediated genetic transformation of FEC of cassava cultivar TME14 and histochemical GUS assays of transformed and non-transformed tissues. (A)** *Agrobacterium*-infected FEC proliferating on selective medium, **(B)** developing embryos on embryo induction medium, **(C)** maturing cotyledonary-stage embryos on shoot induction medium, **(D)** germination of cotyledonary-stage embryos on shoot induction and elongation medium, **(E)** transgenic plantlets regenerated on selective medium, **(F)** transient expression of *gus*A gene in FEC tissues 3 days after co-cultivation with *Agrobacterium*, **(G)** no expression of *gus*A gene in non-transgenic plant, **(H)** stable expression of *gus*A gene in transgenic plant.

Following co-inoculation with *Agrobacterium*, transformed FEC proliferated into small clusters of pale yellow colored resistant calli on paramomycin selection media (Figure 4A). Non-transformed calli stopped growing and turned whitish. Clusters of transformed FEC started developing into somatic embryos after transfer to MSN media (Figure 4B). After six cycles of 10 days each on MSN, FEC gave rise to a total of 713 putatively transformed cotyledonary-stage embryos from three independent experiments (Table 3). After two to three transfers to shoot induction and elongation media (CEM), cotyledonary-stage embryos gave rise to shoots (Figure 4C–E). About 75–82% cotyledon-stage embryos formed shoots (Table 3). It is evident from this study that modification or optimization of the shoot stimulation protocol and more frequent transfers to CEM improved shooting efficiencies. No significant difference (*p* ≤ 0.05) in transformation efficiency was observed among the three experiments performed using different FEC lines. This contributes to the flexibility of the methodology, allowing induction and production of FEC lines to be initiated at different times.

Approximately 70–80 transgenic lines per ml SCV (equivalent to 20 FEC clusters) were regenerated for cultivar TME14 in about 4–5 months from the time of *Agrobacterium* inoculation of FEC. This transformation frequency is significantly higher from previous reports. Bull et al. (2009) reported production of up to 50 transgenic plants after *Agrobacterium*-mediated transformation of 100 clusters of FEC of model cultivar 60444. Zainuddin et al. (2012) reported 7–17 transgenic lines per 18 clusters of FEC of TME cassava landraces. Chetty et al. (2013) reported 23 transgenic lines per 100 clusters of FEC of cassava cultivar T200. Taylor et al. (2012) obtained 14.3–28.6 transgenic plants per cm^3^ of SCV of FEC of model cultivar 60444. Comparison of transformation frequency obtained for the model cultivar 60444, TME landraces and farmer-preferred cultivars in previous reports demonstrated that transformation efficiency reported here is significantly (*p* ≤ 0.05) higher suggesting that the optimized transformation protocol is robust and high-throughput. The lack of significant variation in transformation efficiency between independent experiments and the lack of failed experiments also adds to the utility of the method as reliable research tool.

This optimized protocol opens the doors to the introduction of desirable traits like virus resistance into TME14. To test the adaptability of this protocol, transformation and regeneration capabilities in further cassava transformation experiments were performed for virus resistance (data not shown). About 1100 transgenic lines were regenerated from a total of 10 ml of SCV of FEC in four independent transformation experiments. This suggests that the optimized and validated transformation procedure is reproducible and highly efficient in comparison to previous protocols reported (Bull et al., 2009; Taylor et al., 2012; Zainuddin et al., 2012). Our results suggest that the protocol presented here is useful for introducing functional genes into cassava cultivar TME14.

### Histochemical GUS analysis of transgenic lines

Expression of the *gus*A-intron gene is a reliable indicator of plant transformation, since the gene only can express efficiently in plant cells but not in *Agrobacterium* (Vancanneyt et al., 1990). Transient GUS expression assay 3 days after co-cultivation of FEC tissues showed blue coloration confirming transient expression of the reporter gene in friable embryogenic cells (Figure 4F). A uniform blue coloration was observed in all plants tested from randomly selected transgenic lines, confirming stable expression of *gusA* gene throughout the plant indicating uniform transformation had occurred (Figure 4H). No blue coloration was observed in non-transformed regenerated plants (Figure 4G).

### Molecular analysis of transgenic lines

**Figure 5:**
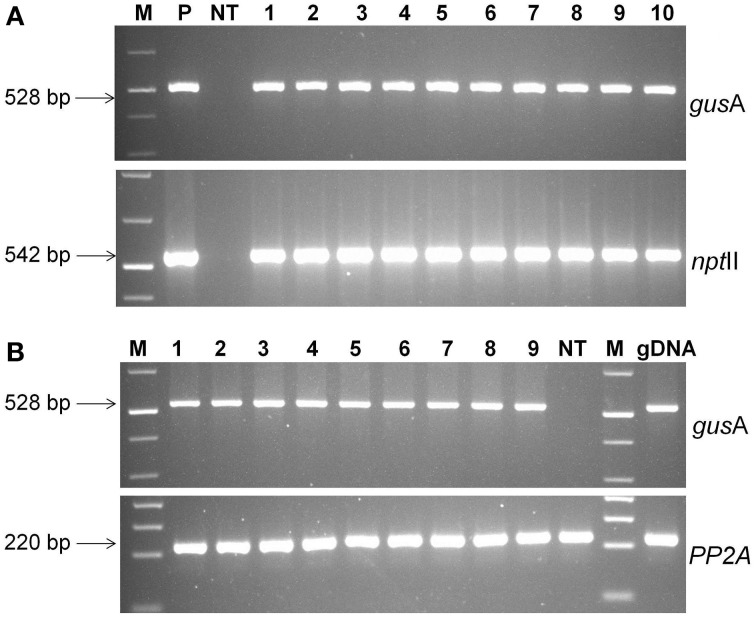
**Molecular analysis of transgenic plants. (A)** PCR analysis of putative transgenic lines of cassava using *gus*A and *npt*II gene specific primers. Lanes M; 1 kb plus molecular marker (Fermentas), P; pCAMBIA2301 plasmid control; NT, non-transgenic regenerated control plant; 1–10, transgenic lines regenerated, (**B)** RT-PCR analysis of transgenic cassava lines using *gusA* and *PP2A* gene specific primers. Amplified RT-PCR product designations were shown on the right, and products sizes were shown on the left. M, molecular weight marker; 1–9, different transgenic lines and NT, non-transgenic control plant.

The presence of transgene in paramomycin resistant regenerated putative transgenic plants was confirmed by PCR analysis. The 528 bp of *gus*A and 542 bp of *npt*II genes were successfully amplified from all the transgenic lines confirming presence of the gene in plant's genome (Figure 5A). No amplification product was detected in non-transgenic control plants.

To determine stable integration of transgene into the plant's genome, Southern blot analysis was performed on randomly selected PCR-positive transgenic lines and a non-transgenic control using a DIG-labeled DNA probe corresponding to the *gus*A gene. All selected plants showed hybridization signals confirming the genomic integration of the transgene (Supplementary Figure 2). Different sizes of bands from the Southern blot experiment represented that the insertion of T-DNA was on different location of the genome. No hybridization was observed in the non-transgenic control plant.

The expression of *gus*A gene was confirmed by RT-PCR analysis. An expected 528 bp fragment was amplified from the cDNA products of transgenic lines, but was absent in non-transgenic plants (Figure 5B). Specific *PP2A* transcript amplification was detected from all plants as an internal control for cDNA synthesis. These results demonstrated that *gusA* transgene was successfully incorporated into the genome of regenerated plants and functionally expressed transgenic cells.

## Conclusions

We report here the development of a high-throughput system for *Agrobacterium*-mediated transformation and regeneration of an important farmer preferred cassava cultivar “TME14” using FEC. We have made improvements in tissue culture and regeneration protocols that will make the production of transgenic cassava plants more efficient than the protocols described in earlier reports. Transformed FEC generated plants with high efficiency, at 70–80 transgenic lines per ml of SCV. This high-throughput method could be used for studying gene manipulation and transferring new traits into farmer preferred cassava cultivar TME14. We have already generated over a thousand transgenic TME14 lines using this protocol as a step toward developing CBSD resistance. This protocol can be applied to other farmer preferred cultivars of cassava. We have tested the adaptability of this protocol in another farmer preferred cultivars like “Albert” and “Mkombozi,” which were found to be difficult to transform in our previous study (Nyaboga et al., 2013). The factors which favored somatic embryogenesis in TME14, also able to produce FEC in “Albert” and “Mkombozi.” By applying improved transformation protocol for TME14, the FEC of these two cultivars were transformed using *gus*A reporter gene and transgenic lines were generated with transformation efficiencies similar to TME14, suggesting that the *Agrobacterium*-mediated transformation protocol developed for TME14 can be adapted to other important cultivars for crop improvement.

### Conflict of interest statement

The authors declare that the research was conducted in the absence of any commercial or financial relationships that could be construed as a potential conflict of interest.

## Supplementary material

Supplementary Figure 1 **Schematic representation of gene construct used for genetic transformation of cassava**. The T-DNA region of construct pCAMBIA2301 with *gus*A and *npt*II genes. *npt*II, coding region of the *neomycin phosphotransferase* gene; NOS, nopaline synthase terminator; intron-*gus*A, β-*glucuronidase* containing intron; MCS, multiple cloning site; 35SP, CAMV35S promoter.

Supplementary Figure 2 **Southern analysis of genomic DNA of transgenic lines and non-transgenic control plant (NT) using *Hind*III restriction enzyme**.
