# Supplementary figures and images for: Factors influencing somatic embryogenesis, regeneration, and Agrobacterium-mediated transformation of cassava (Manihot esculenta Crantz) cultivar TME14
Source: Front Plant Sci. 2015 Jun 10;6:411. doi: 10.3389/fpls.2015.00411 (PMC4461822; doi:10.3389/fpls.2015.00411)

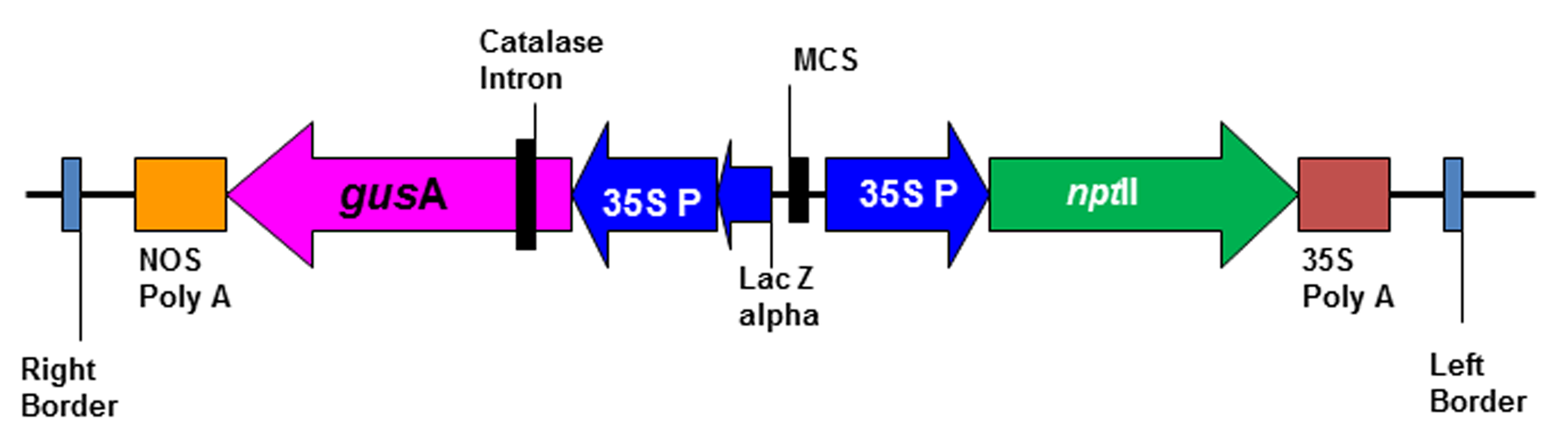

Supplement: Supplementary Figure 1 — Schematic representation of gene construct used for genetic transformation of cassava. The T-DNA region of construct pCAMBIA2301 with gusA and nptII genes. nptII, coding region of the neomycin phosphotransferase gene; NOS, nopaline synthase terminator; intron-gusA, β-glucuronidase containing intron; MCS, multiple cloning site; 35SP, CAMV35S promoter. [file Image1.TIF]

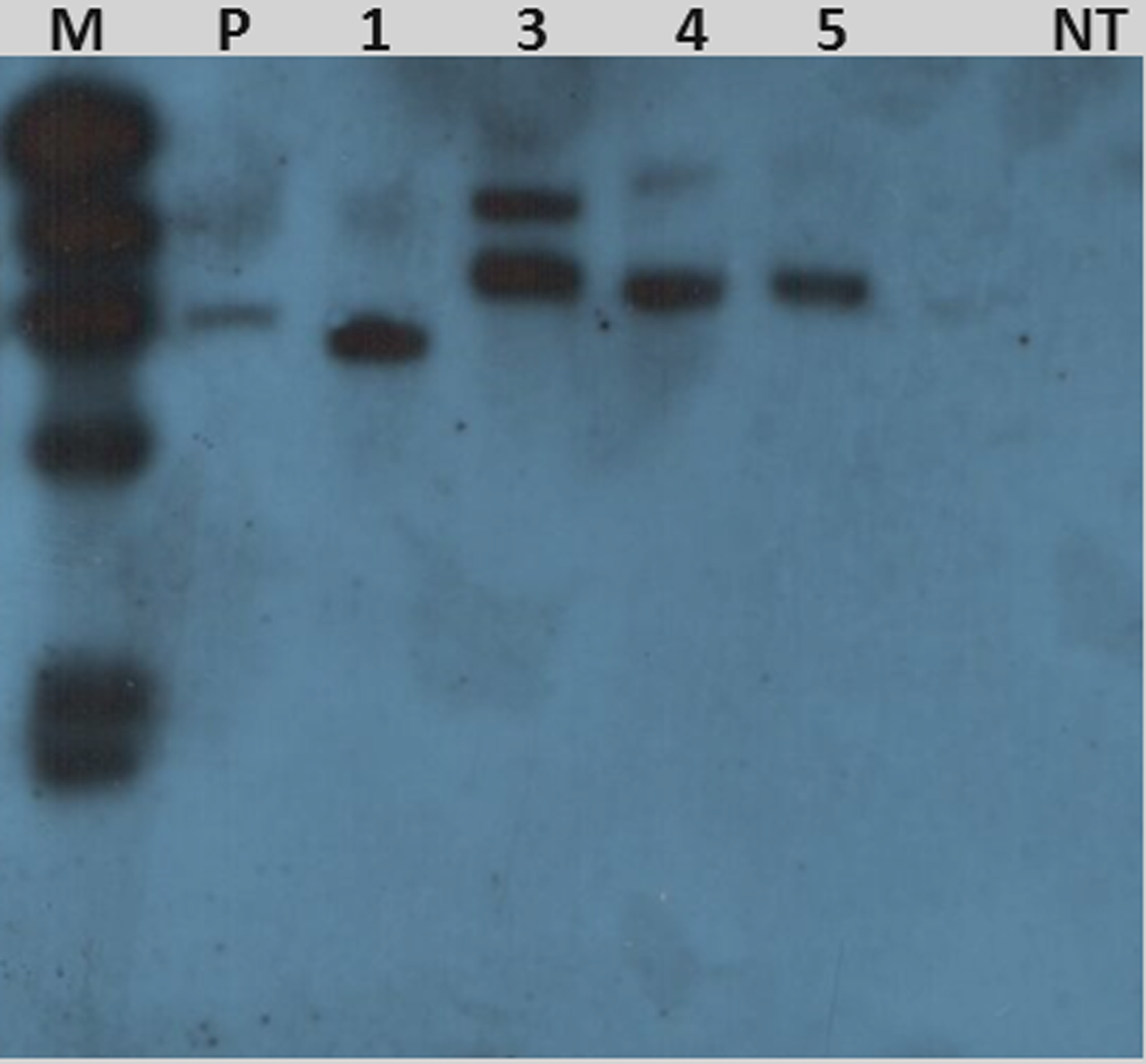

Supplement: Supplementary Figure 2 — Southern analysis of genomic DNA of transgenic lines and non-transgenic control plant (NT) using HindIII restriction enzyme. [file Image2.TIF]
