# Supplementary material for: Factors influencing somatic embryogenesis, regeneration, and Agrobacterium-mediated transformation of cassava (Manihot esculenta Crantz) cultivar TME14
Source: Front Plant Sci. 2015 Jun 10;6:411. doi: 10.3389/fpls.2015.00411 (PMC4461822; doi:10.3389/fpls.2015.00411)
Supplement: Supplementary file 3 [file Table1.PDF]

Supplementary Table 1:

Composition of media used in tissue culture and transformation experiments.

Name of medium	Composition
Basic shoot culture medium (CBM)	1 × MS[a] salts with vitamins, 2 μM $CuSO4$, 2% sucrose, 0.3% Gelrite, pH 5.8
Axillary bud enlargement medium (CAM)	1 × MS salts with vitamins, 2 μM $CuSO4$, 10 mg/l BAP (6-benzylamino purine), 2% sucrose, 0.8% Noble agar, pH 5.8
Somatic embryo induction medium (CIM-MS)	1 × MS salts with vitamins, 2 μM $CuSO4$, 12 mg/l picloram, 2% sucrose, 0.8% Noble agar, pH 5.8
Somatic embryo induction medium (CIM-DKW)	1 × DKW[b] salts with vitamins, 2 μM $CuSO4$, 12 mg/l picloram, 2% sucrose, 0.8% Noble agar, pH 5.8
Friable embryogenic calli medium (GD)	1 × GD[c] salts with vitamins, 12 mg/l picloram, 2% sucrose, 0.8% Noble agar, pH 5.8
Somatic embryo emerging medium (MSN)	1 × MS salts with vitamins, 1 mg/l NAA (Naphthaleneacetic acid), 2% sucrose, 0.8% Noble agar, pH 5.8
Shoot elongation medium (CEM)	1 × MS salts with vitamins, 2 μM $CuSO4$, 0.4 mg/l BAP, 2% sucrose, 0.8% Noble agar, pH 5.8

[a]MS, Murashige and Skoog (1962); [b]DKW, Driver and Kuniyuki (1984); [c]GD, Gresshoff and Doy (1974)
